# The development of a systematic ultrasound protocol facilitates the visualization of foreign bodies within the canine distal limb

**DOI:** 10.3389/fvets.2023.1298072

**Published:** 2023-12-20

**Authors:** Ebony Schoenfeld, Martin Combs, Esther Callcott, Kieri Jermyn, Randi Rotne

**Affiliations:** School of Agricultural, Environmental and Veterinary Services, Charles Sturt University, Wagga Wagga, NSW, Australia

**Keywords:** ultrasonography, canine interdigital grass seed foreign body, musculoskeletal ultrasonography, canine distal limb, ultrasonographic protocol, vegetal foreign body

## Abstract

Ultrasonography is an excellent investigative tool that can assist with the diagnosis of soft tissue conditions. In human medicine, ultrasonography is a fundamental diagnostic tool for the investigation of suspected vegetal foreign bodies (VFB), with protocol-based ultrasonography providing increased accuracy compared to lesion-focused examinations. Protocol-based ultrasonography is an emerging tool within the veterinary field, however, compared to human medicine is not routinely employed. The objective of this study was to develop a systematic ultrasound protocol to examine the distal limb for the visualization of vegetal foreign bodies (SUEDVEG). A 12 MHz linear and an 18 MHz high-frequency small-footprint linear array transducer was used on cadaver forelimbs (*n* = 6) and hindlimbs (*n* = 6) with images obtained from three common foreign body locations within the distal limb; 1; the interdigital webbing, 2; the palmar/plantar aspect of the phalanges and metacarpus and 3; the dorsal region of the phalanges and metacarpus. From these images, a 13-step systematic musculoskeletal protocol was developed and utilized on eight clinical cases or 10 limbs that had signs typical of distal limb VFB to preliminarily validate the proposed method. Vegetal foreign bodies were successfully identified and retrieved in seven (*n* = 8) clinical cases with method steps 9 and 11 (orthogonal views) identifying the majority of VFBs. The described ultrasound method appears highly useful for visualizing soft tissue locations of the canine distal limb known for tracking foreign bodies. Further studies are required to validate the described systematic examination method as the preferred clinical protocol over currently used lesion-focused exploration techniques.

## 1 Introduction

Ultrasonography is a practical and inexpensive tool that can be used to aid the diagnosis of musculoskeletal disease (1). Ultrasonography is indicated for the assessment of musculoskeletal conditions such as diffuse or focal soft tissue swelling and can aid therapeutic interventions such as foreign body retrievals (2, 3). Musculoskeletal ultrasonography, however, can often be challenging due to similarities in tissue echogenicity and the requirements of specific transducer angulation to visualize certain anatomy (4). It is because of this that ultrasonography has been identified as an advanced veterinary skill and low clinician confidence has been linked to this investigative technique (5–7).

The most common foreign body type observed within veterinary practice tends to be organic. Migrating vegetal foreign bodies such as the grass seed, represents 2 % of cases seen by small animal veterinarians within the Riverina, New South Wales, Australia (8). Known for their sharp tip and multiple barbs, the spindle-shaped grass seed reliably attaches to the hair or wool of an animal. This shape along with regional movement, enables the seed to track and burrow into the skin of the animal. Seed migration then occurs through the tissue planes in a unidirectional path; propelled forward by the multiple barbs located at the seed base (9).

Vegetal foreign bodies can be located anywhere within the body. The most commonly observed location for VFBs is the ear (46.7%) followed by the interdigital webbing (14.8%) (8, 10). Vegetal foreign bodies within the distal limb; specifically the interdigital webbing, can often be difficult to diagnose due to the non-specific clinical presentation (8, 10, 11). Furthermore, the grass seed penetrates the interdigital webbing and can traverse through the different tissue planes within the distal limb unidirectionally, meaning they can have varying clinical signs (9). Patients with VFBs can present for lameness, draining tracts within the webbing, inflammation, cellulitis and/or swelling of a single (thoracic or pelvic) limb (12). These varying clinical presentations and the radiolucent nature of VFBs, have led to the use of focused ultrasound examinations in referral practice (13).

Vegetal foreign bodies occur more commonly in dogs than cats (14, 15). Cases typically occur during summer when compared to winter with male dogs at greater risk in both Europe (16) and Australia (8). Medium coat length breeds are at higher risk of vegetal foreign bodies with working dogs and security dogs over-represented (8). Specific breeds commonly presenting include spaniels, border collies, Staffordshire terriers, golden retrievers, Australian kelpies, Labrador retrievers and Shih Tzus (8, 16, 17).

Ultrasonography is a proven, beneficial tool for the diagnosis of VFBs. Analyzing 46 papers, Caivano et al. (11) completed a systematic review that observed several commonalities between VFB cases reported. Microconvex and linear probes were consistently used to identify VFBs with transducer frequency dependent on the depth of the region of interest. Additionally, sonographers used both the identification of the VFB and/or its secondary lesions to localize and remove the migrating VFB. Furthermore, the appearance of a grass seed VFB on ultrasound was distinctive regardless of size or location. A grass seed VFB was commonly defined as a hyperechoic, spindle-shaped structure surrounded by a hypoechoic region. It was noted, however, that while the size and location of the VFB did not impact the ultrasonographic appearance, it did impact the ease of visualization and retrieval choice (11). Furthermore, the characteristic ultrasonographic appearance of grass seed VFBs has also been shown to have the potential to differentiate between interdigital abscessation secondary to VFBs and interdigital furunculosis (17).

Ultrasonographic descriptions of the canine distal limb have been a focus of recent research. Ultrasound has been used to assess tenosynovitis of the abductor pollicis longus muscle (18) and describe the ultrasonographic anatomy of the carpal joint in the Border Collie (19). One study of particular interest, has described the ultrasonographic appearance of the dorsal region of the canine carpus (20). When examining the distal third of the antebrachium, González-Rellán et al. (20) observed that having a systematic protocol enabled both a complete and organized examination of the region.

The use of established systematic musculoskeletal protocols within the veterinary industry are limited and therefore extrapolations need to be made from human medicine. In the human emergency department, ultrasonography has been observed to assist doctors with the speed and accuracy of patient diagnosis, resulting in faster, more appropriate treatment (21–23). Although focused ultrasound examinations have been found to be rapid and specific, a protocol-based approach is more accurate for the detection of VFBs (24). Jamadar et al. (24), observed that focused ultrasonographic examinations can result in false negatives due to confounding factors such as referred pain, diffuse symptoms and abnormalities occurring outside the affected area. The benefits of protocol-based sonography are known within the human medical industry and scanning protocols for the shoulder, elbow, carpus and hand have been developed to ensure that all essential views are routinely obtained to evaluate anatomical locations where musculoskeletal conditions commonly occur (25).

This study describes a new method that can be applied in general veterinary practice to examine the canine distal limb sonographically. The developed SUEDVEG method, focused on potential locations for VFBs in the distal limb to allow for accurate visualization of soft tissue structures. In this investigation, two differing transducers were utilized to examine the distal limb prior to it being utilized on eight clinical cases in veterinary practice.

## 2 Methods

### 2.1 Musculoskeletal ultrasound protocol design and development

Canine cadavers were collected and stored frozen at Charles Sturt University (animal ethics approval: A23561). Limbs were assessed for visible iatrogenic or pathological changes distal to the stifle/elbow and were excluded from this study if present. A sample of canine forelimbs (*n* = 6) and hindlimbs (*n* = 6) were collected. Forelimbs and hindlimbs were amputated parallel to the scapula and at the acetabulofemoral joint, respectively, before being stored in a maximum – 18°C walk-in freezer.

Two like limbs (i.e., two fore- or two hindlimbs) were thawed at room temperature overnight. The following morning, the limbs were inspected to ensure they were completely thawed, and the pliability was assessed to ensure no excessive freezer burn (preventing limb flexion). Each limb was towel-dried and clipped using size 40 blades (Aesculap, Pennsylvania, United States). Fore- and hindlimbs were carefully clipped to the proximal third radius or distal third tibia respectively, before being decontaminated with a chlorhexidine scrub brush (BD E-Z Scrub^™^, Becton Dickinson, New Jersey, United States) and hand soap. Each limb was then lightly misted with a water mixture containing 10% glycerine (Vetsense Animal Health, Mulgrave, New South Wales, Australia) and 1% chlorhexidine (Henry Schein^®^, Mascot, New South Wales, Australia) to maintain moisture before undergoing a systematic sonographic investigation using a Logiq S8 ultrasound (GE Healthcare, Illinois, United States) and two transducers.

Two transducers were used to complete the systematic protocol. The limbs were examined using a Logiq 12 MHz linear transducer (GE Healthcare, Illinois, United States) and a Logiq 18 MHz high frequency small-footprint linear array transducer (GE Healthcare, Illinois, United States). During these systematic examinations, comparisons between the two transducer types was explored with depth, focal point, dynamic range, and frequency altered to determine the optimal settings for each ultrasound image.

**Figure 1:**
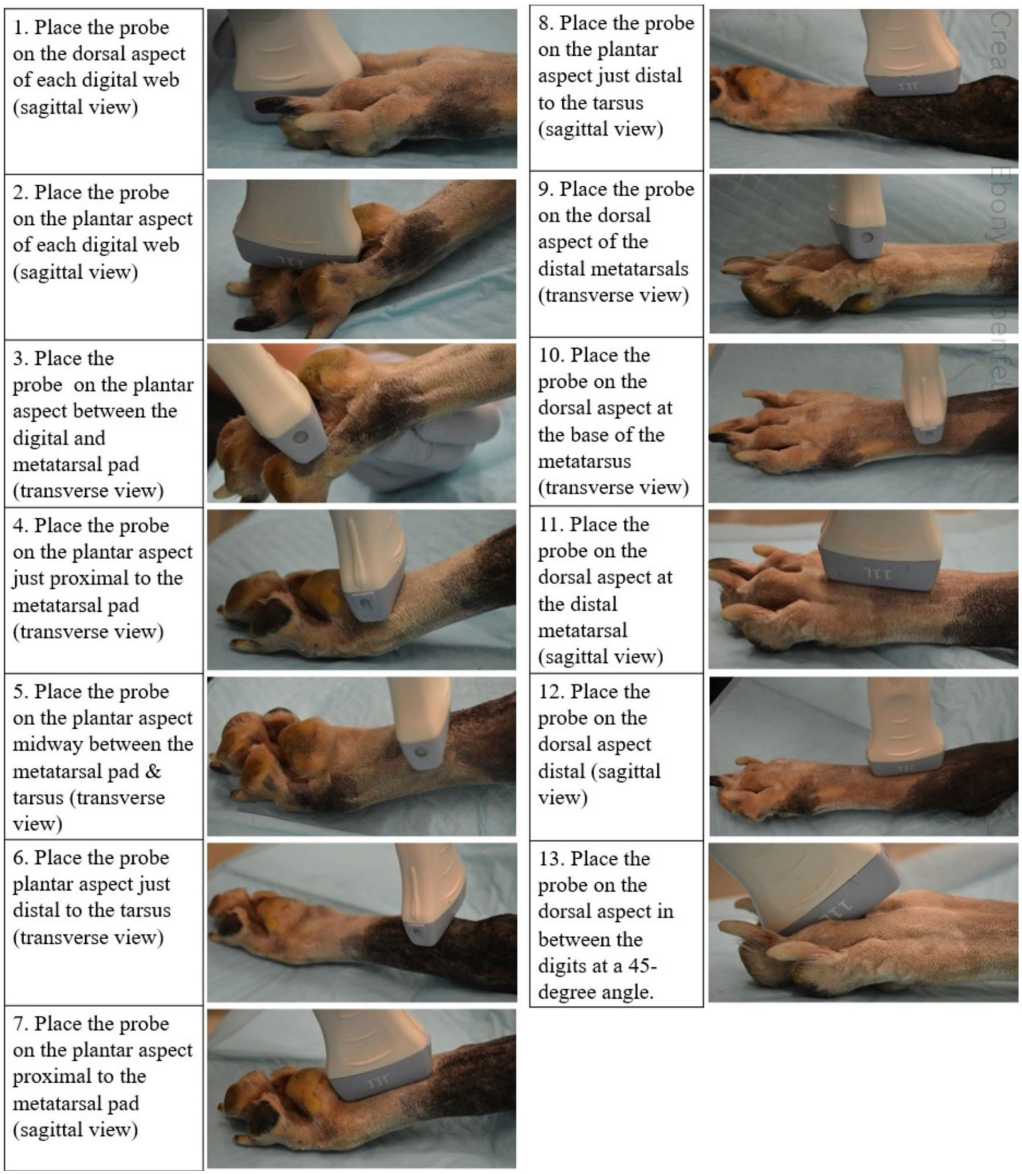
A complete visual of the 13-step ultrasonographic protocol on the canine distal hindlimb.

Three regions of the canine distal limb were systematically assessed (Figure 1). Firstly, the interdigital webbing was examined. The transducer was placed sagittally on both the dorsal and palmar/plantar aspect of each digital web (Step 1 and 2). The probe was then placed transversely on the palmar/plantar aspect between the digital and metatarsal pad (Step 3).

Next, the palmar/plantar region of the distal limb was examined. The transducer was placed transversely on the palmar/plantar aspect just proximal to the metacarpal/metatarsal pad (Figure 1; Step 4). The transducer, in the same transverse field, was then swept proximally to the distal tarsus (Step 5 and 6). The same locations on the palmar/plantar aspect were then reassessed in the orthogonal (sagittal) view (Step 7 and 8). The probe was also placed on the dorsal aspect of the limb in between the digits at a 45-degree angle (Step 13).

Finally, the dorsal aspect of the distal limb was assessed. The probe was placed on the distal metatarsals/metacarpals to obtain a transverse view before it was swept proximally to the base of the metatarsals/metacarpals (Figure 1; Step 9 and 10). Once images were obtained within these regions, the transducer was placed sagittal on the same two locations to provide orthogonal views (Step 11 and 12).

This systematic canine distal limb ultrasound method was developed using an iterative process that involved experienced clinicians and anatomists identifying key ultrasound examination locations to optimize and standardize ultrasound image acquisition. Following initial image acquisition, image quality, examination sites and overall imaging protocol (number of examination sites, images collected at each site and the imaging sequence) were further refined to develop the 13 step SUEDVEG protocol. The ultrasound method was then repeated on several limbs from different canine cadavers to assess the repeatability of the established method.

### 2.2 Application of the systematic ultrasound protocol

During November 2020, the ultrasound protocol was used on patients with a suspected VFB (animal ethics approval A20310). Authors attended local (Riverina, New South Wales, Australia) veterinary clinics to assess patients with suspected distal limb VFBs and complete the ultrasonographic examination on the limb. Once the SUEDVEG protocol was completed and a VFB was detected on ultrasound, retrieval was undertaken using ultrasound guided techniques.

Patient signalment and history was obtained from the admitting veterinarian. Dogs were included in the study based upon clinical signs; specifically, the presence of localized focal subcutaneous swelling distal to the carpus/manus and/or an interdigital draining tract. If additional information was required, the owner was contacted, and the patient history file viewed prior to anesthesia. Each patient was examined and then anesthetized as per the clinic’s standard protocol. Affected limb(s) were clipped using size 40 blades. Fore- and hindlimbs were clipped from the interdigital webbing to the proximal third radius or distal third tibia using Shear Magic^®^ Rocket 4,500 and Saphir style (Heiniger, Bibra Lake, Western Australia, Australia) clippers, respectively. The limb was then decontaminated with BD E-Z chlorhexidine scrub.

Each affected limb underwent the previously developed 13 step SUEDVEG protocol. All steps were completed regardless of whether a VFB was found within the earlier steps. When a VFB was found, the location and protocol steps used to detect it were recorded. Clients were advised to return for a follow-up appointment should clinical signs persist 7 days post-retrieval. Resolution was defined as patients not requiring a follow-up appointment in 7 days post procedure with the client attending at least another unrelated appointment within 12 months of VFB retrieval (i.e., they remained a client of the clinic).

## 3 Results

### 3.1 Musculoskeletal ultrasound protocol design and development

**Figure 2:**
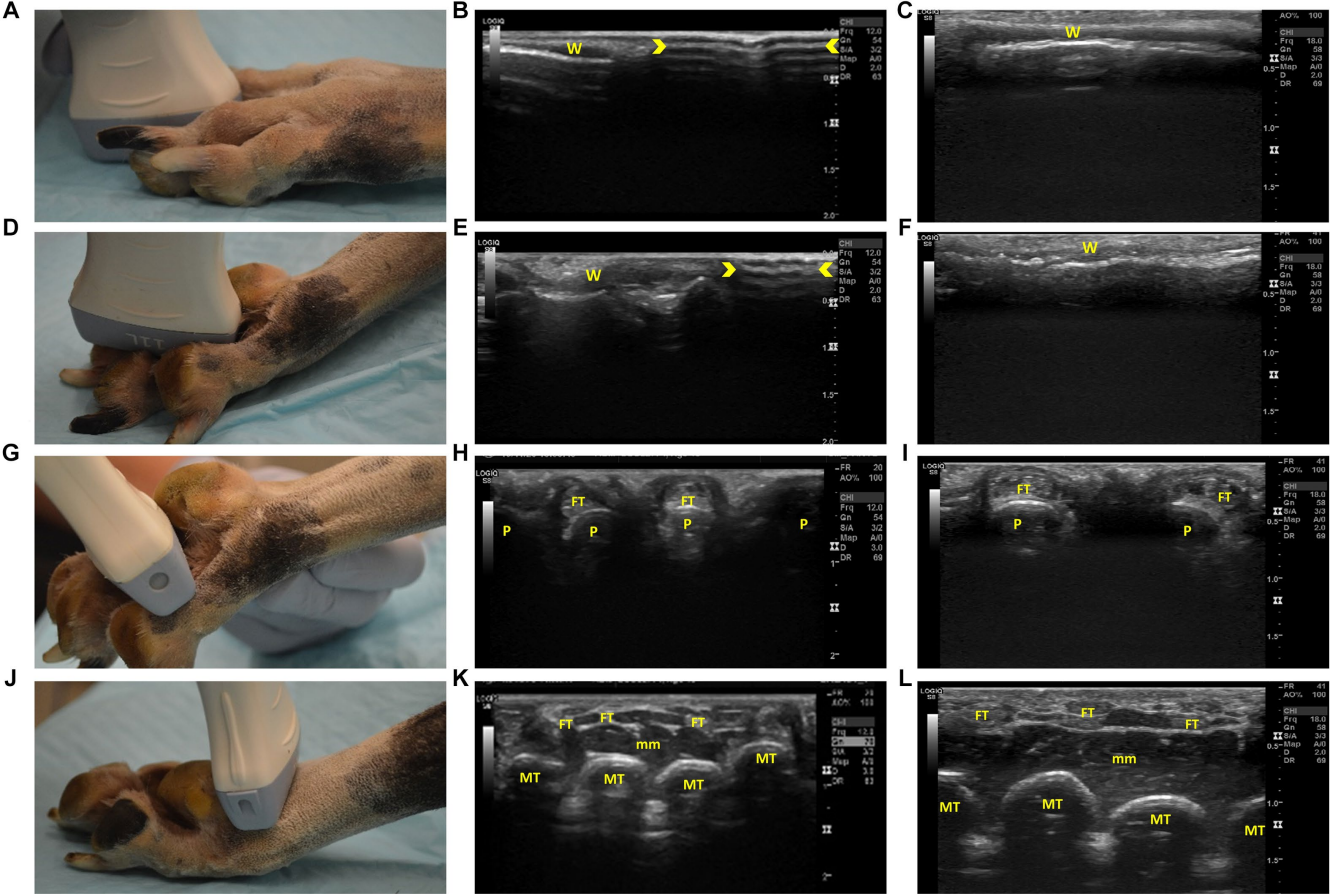
Steps 1–4 of the ultrasonographic protocol with visual representation of the location and ultrasonographic images obtained using the linear and high frequency small-footprint linear array (HFSLA) transducer. **(A)** Step 1: The probe was placed on the dorsal aspect of each digital web to obtain a sagittal view; **(B)** Step 1 ultrasound image using the linear transducer; **(C)** Step 1 ultrasound image using the HFSLA transducer; **(D)** Step 2: The probe was placed on the plantar aspect of each digital web to obtain a sagittal view; **(E)** Step 2 ultrasound image using the linear transducer; **(F)** Step 2 ultrasound image using the HFSLA transducer; **(G)** Step 3: The probe was placed on the plantar aspect between the digital and metatarsal pad to obtain a transverse view; **(H)** Step 3 ultrasound image using the linear transducer.; **(I)** Step 3 ultrasound image using the HSFLA transducer; **(J)** Step 4: The probe was placed on the plantar aspect just proximal to the metatarsal pad to obtain a transverse view; **(K)** Step 4 ultrasound image using the linear transducer; **(L)** Step 4 ultrasound image using the HFSLA transducer. Annotations: 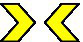, image dropout; W, interdigital webbing; P, phalange; FT, flexor digitum profundus tendon; MT, metatarsal; mm, muscle layers.

**Figure 3:**
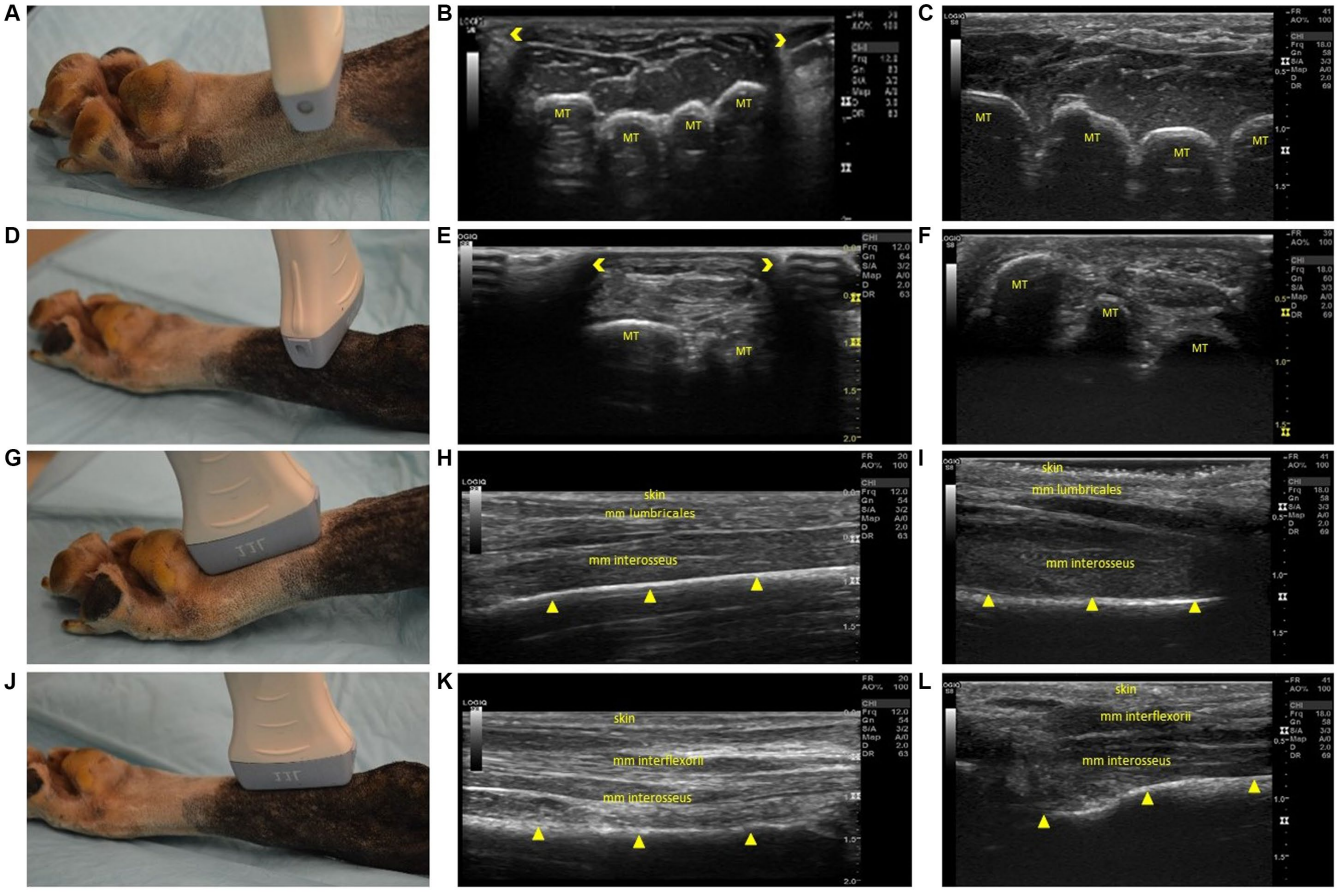
Steps 5–8 of the ultrasonographic protocol with visual representation of the location and ultrasonographic images obtained using the linear and high frequency small-footprint linear array (HFSLA) transducer. **(A)** Step 5: The probe was placed on the plantar aspect midway between the metatarsal pad and tarsus to obtain a transverse view; **(B)** Step 5 ultrasound image using the linear transducer; **(C)** Step 5 ultrasound image using the HFSLA transducer; **(D)** Step 6: The probe was placed on the plantar aspect just distal to the tarsus to obtain a transverse view; **(E)** Step 6 ultrasound image using the linear transducer; **(F)** Step 6 ultrasound image using the HFSLA transducer; **(G)** Step 7: The probe was placed on the plantar aspect proximal to the metatarsal pad to obtain a sagittal view; **(H)** Step 7 ultrasound image using the linear transducer; **(I)** Step 7 ultrasound image using the HFSLA transducer; **(J)** Step 8: The probe was placed on the plantar aspect just distal to the tarsus to obtain a sagittal view; **(K)** Step 8 ultrasound image using the linear transducer; **(L)** Step 8 ultrasound image using the HFSLA transducer. Annotations: 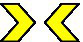, image dropout; MT, metatarsal; 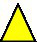, bone interface.

**Figure 4:**
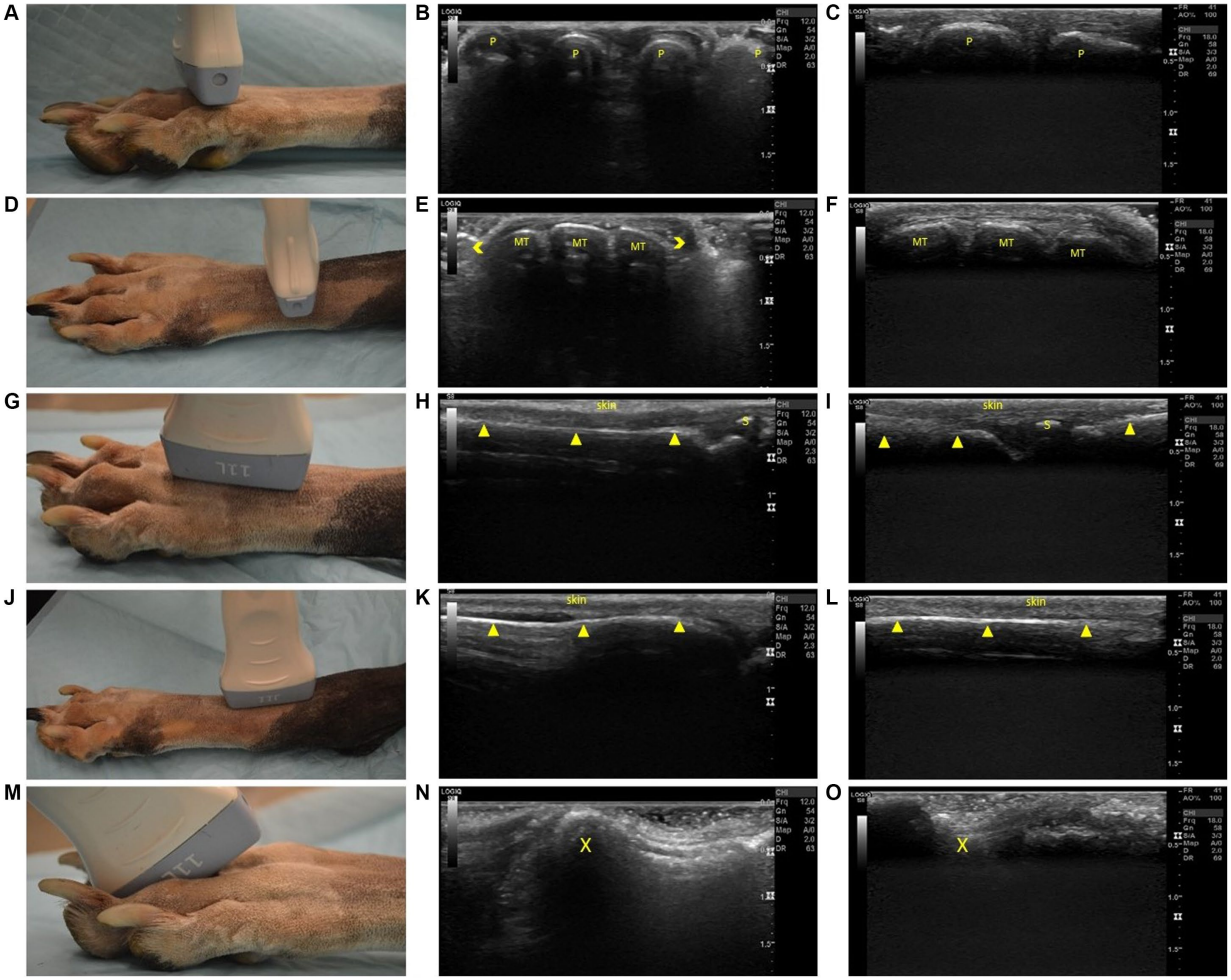
Steps 9–13 of the ultrasonographic protocol with visual representation of the location and ultrasonographic images obtained using the linear and high frequency small-footprint linear array (HFSLA) transducer. **(A)** Step 9; The probe was placed on the dorsal aspect at the distal metatarsals; viewing the junction between the head of the metatarsal bone and proximal phalange transversely; **(B)** Step 9 ultrasound image using the linear transducer; **(C)** Step 9 ultrasound image using the HFSLA transducer; **(D)** Step 10: The probe was placed on the dorsal aspect at the base of the metatarsus to obtain a transverse view; **(E)** Step 10 ultrasound image using the linear transducer; **(F)** Step 10 ultrasound image using the HFSLA transducer; **(G)** Step 11: The probe was placed on the dorsal aspect at the distal metatarsal; fanning for a sagittal view of the interosseous muscles; **(H)** Step 11 ultrasound image using the linear transducer; **(I)** Step11 ultrasound image using the HFSLA transducer; **(J)** Step 12: The probe was placed on the dorsal aspect distal to the tarsus for a sagittal view; **(K)** Step 12 ultrasound image using the linear transducer; **(L)** Step 12 ultrasound image using the HFSLA transducer; **(M)** Step 13: The probe was placed on the dorsal aspect in between the digits at a 45-degree angle; **(N)** Step 13 ultrasound image using the linear transducer; **(O)** Step 13 ultrasound image using the HFSLA transducer. Annotations: 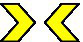, image dropout; MT, metatarsal; 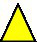, bone interface P, phalange, X, main digital pad visualization; s, digital sesamoid.

A 12 MHz linear transducer and an 18 MHz high frequency small-footprint linear array transducer were used to complete the ultrasound examination. For both transducers, the region of interest was best seen when the depth was set between one and three centimeters. In addition, the best resolution was obtained with two focal points; one at 0.5 and at 1 centimeter depth. Dynamic range varied between limbs and anatomical locations as required with 63 dB found to be an ideal starting point for both transducers. Figures 2–4 compare the 12 MHz linear transducer and the 18 MHz high frequency small-footprint linear array transducer (HFSLA) images of the hindlimb.

The best images obtained in all steps, occurred when the limb was completely clipped, and the transducer was perpendicular to the region being ultrasounded. Perpendicular alignment resulted in increased transducer contact, resulting in fewer ultrasonographic artifacts. This was especially important when visualizing between the interdigital webbing and digital pads. Limb flexion, fanning and rocking were also used to improve image quality.

The protocol systematically assessed the limb in a distal to proximal direction. Starting at the most distal aspect of the limb, the thin (0.2–0.4 cm) epidermal and dermal layers of the interdigital webbing could be seen (Figures 2B,C). The large linear transducer size (Figure 2B) caused image drop out due to poor contact with the body surface which meant that the webbing was just visible on the left half of the ultrasound image. Comparatively, the HFSLA had complete skin contact and therefore an ultrasound image was visible on the entire screen (Figure 2C).

Continuing with the assessment of the interdigital webbing, Step 2 (Figure 2D) had several similar findings to Step 1. Fanning in both steps assisted with the visualization of the areas adjacent to the digital bones, however, poor contact was once more an issue with the linear transducer resulting in image drop out (Figure 2E). Additionally, a true sagittal view was difficult to obtain in Step 2 due to the regional anatomy (specifically the main digital pad and digit placement) resulting in the smaller HFSLA having better quality images due to the better contact between the probe and limb.

The second region assessed ultrasonographically, was the plantar aspect of the distal limb (Figures 2G–L, 3A–F). When assessing the webbing between the pads, pressure on the dorsal surface, flexion of the digits around the probe and additional ultrasound gel was required to increase contact. Improved contact enabled visualization of the phalanges and the corresponding flexor digitum profundus tendon when using both the HFSLA and linear transducers (Figures 2H,I). Four phalanges in comparison to two were visible with the linear transducer due to contact with all four digits. In addition, limb flexibility, patient size, and probe contact, made fanning extremely difficult and unrewarding at Step 3. However, fanning the probe in Step 4 enabled some visualization under the main digital pad which provided supplemental information to the imaging obtained in Step 13 (Figure 4M).

Fanning, rocking and compression were essential for image quality in Step 5 (Figure 3A) and 6 (Figure 3D). Using these three techniques enabled complete visualization of each region being examined. Additionally, it should be noted that image drop out was observed using the linear transducer (Figures 3E,H). Regardless of transducer type, it was difficult to distinguish between the muscle’s mm interflexorii, proximal mm interosseus and mm adductor digitti located within this region.

For a complete view of the plantar region, several sagittal views (Figure 3) were required. Within Step 8 (Figure 3J), the ultrasound appearance of muscle was like that of all other skeletal muscle. The muscle superimposing the bone, had a hypoechoic background with layered, echogenic striations representing the multiple muscle fiber bundles. Specifically, the mm lumbricales, mm interosseus and mm interflexorii were observed in these two steps. Additionally, acoustic impedance was observed as expected in both the linear and HFSLA transducers preventing visualization past the bone (Figures 3H,I,L,M). To completely visualize the plantar region, images under the metatarsal pad needed to be obtained. In this instance, it was found that when the probe was placed on the dorsal aspect in between the digits at a 45-degree angle (Step 13; Figure 4M), a quality image could be obtained when pressure was applied to the palmar aspect of the metatarsal pad.

The third region assessed was the dorsal surface (Figure 4). Fanning, rocking and flexion of the limb was required to completely visualize the junction between the head of the metatarsal bone and proximal phalanges within Step 9 (Figure 4A). The metatarsals were observed to be shallow (<0.5 cm) in both the linear and HFSLA transducer images at both Step 9 and Step 10. This was because there were no muscles traversing the metatarsals; just the fascia dorsalis pedis for stabilization. Furthermore, partial visualization of the tarsus occurred with both transducers (Figures 4E,F). Only three metatarsals were visible on the linear transducer with image drop out surrounding the region compared to a full screen image of two to three metatarsal using the HFSLA transducer. This was caused by the curvature of the limb and transducer size, respectively.

Sagittal views completed the ultrasound examination of the dorsal aspect of the canine distal limb. Fanning and rocking was once more required at Step 11 and 12 (Figures 4G,J) to enable visualization of the entire tarsus (including metatarsals and interosseous muscles). In both these regions, a shallow (<0.3 cm) image containing no muscles; just skin and bone was obtained. Additionally, it was noted that the dorsal sesamoid bone could observed at the metatarsal-proximal phalange joint in the sesamoid fossa in Step 11 (Figures 4H,I).

**Figure 5:**
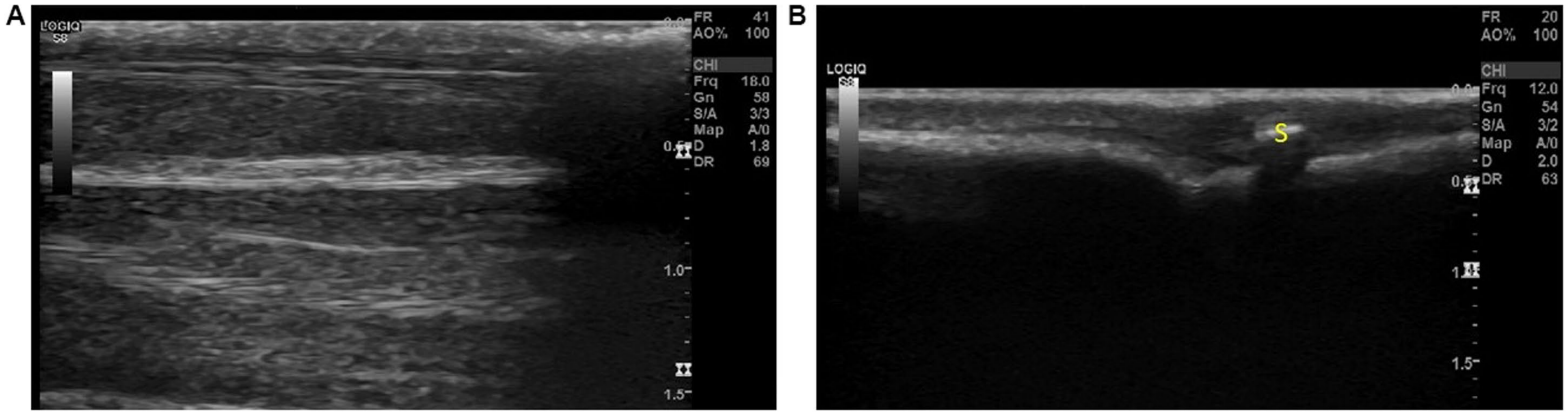
The two views observed with step 11; **(A)** Between the metacarpals/metatarsals the interosseous muscles can be observed on ultrasound and **(B)** Over the metacarpals/metatarsals, a shallow image of the bone surface can be visualized. The digital sesamoid was observed over the joint and could be mistaken for a foreign body. Annotations: S, digital sesamoid.

When undertaking Step 11, it was observed that two images could be obtained (Figure 5). When parallel to the metacarpals or metatarsals, a very shallow image revealed little to no visible musculature. The bone surface and digital sesamoid were observed at a depth no greater than 0.5 cm (Figure 5B), whereas when placed between the two metacarpals or metatarsals, visualization of the short interossei muscles could be visualized at greater depths (Figure 5A). It was commented that the digital sesamoid could be mistaken for a VFB due to its size and echogenicity.

### 3.2 Application of musculoskeletal ultrasound protocol

The musculoskeletal ultrasound protocol, SUEDVEG, was utilized in eight clinical cases (Table 1). From the clinical cases, one patient (Table 1; Case 7; a six-year-old male entire German Shepherd Dog) presented with clinical signs suspicious of VFBs within three (left fore, left hind and right hind) limbs. Because of this, each affected limb of Case 7 was examined systematically resulting in the ultrasonographic protocol being used 10 times (i.e., 10 limbs were methodologically examined across all cases (*n* = 8)).

**Table 1:** Application of musculoskeletal ultrasound protocol on eight clinical cases.

	Signalment	Affected limb	No. of limbs ultrasounded	Clinical signs	VFB located?	No. of VFBs found	Protocol views	Comments	Follow-up appointment
1	13y MN Maltese Terrier	RF	1	Discrete erythema and swelling on the dorsal surface of the proximal metacarpals (2.5 cm diameter), draining tract between III/IV digits	Yes	1	10, 11 and 12	Identified in Step 10 and confirmed with orthogonal view (step 11)	No
2	4y MN Poodle	RH	1	Furunculosis like lesions and draining tracts between III/IV digits. Mild swelling of all digits	Yes	1	9 and 11	Identified in step 9 and confirmed with orthogonal view (Step 11). Additional two VFB retrieved from furunculosed skin that had not yet penetrated the dermis	No
3	10y ME Jack Russell Terrier	LF	1	Lame for 10 days. Swelling and erythema with draining tract between II/III digits.	Yes	1	9 and 11	Identified in step 9 and confirmed with orthogonal view (Step 11)	No
4	2y ME Staghound	RF	1	Scabbing between IV/V digits; swelling over the digit and proximal metacarpals	No	0	N/A	History of VFB retrieval 3 weeks ago from same limb. Consider VFB remnant hairs or infectious processes secondary to previous VFB.	Antibiotics provided; follow-up appointment not attended
5	2y FN Golden Retriever	LF	1	draining tract between III/IV digits, abscess on IV lateral toe	Yes.	1	1, 9 and 11	Step 11 transducer placement was more distal and included the digital webbing.	No
6	2y MN long haired Jack Russell Terrier	RF	1	draining tract between III/IV digit. Localized swelling proximal to the webbing	Yes	1	9, 11 and 13	Best image obtained from step 13	No
7	6y ME German Shepherd Dog	LF LH RH	3	Visible VFBs between the digits. LF = draining tract between III/IV digits LH = draining tracts between II/III and III/IV digits RH = draining tracts between I/II digits	Yes; from 3 limbs	7	LF = 1, 4, 9, 11 and 13 LH = 1, 3, 9, 11 and 13 RH = 1, 9 and 11	Air artifact develops upon removal of the first VFB. Ensure entire protocol completed prior to retrieval	Antibiotics provided; follow-up appointment not attended
8	5y FN Corgi X Pomeranian	RF	1	Draining tract between IV/V digits. Swelling and erythema proximal to the draining tract	Yes	1	9 and 11	Identified in step 9 and confirmed with orthogonal view (Step 11). Rose thorn removed.	No

VFB; vegetal foreign body, RF; right forelimb, RH; right hindlimb, LF; left forelimb, LH; left hindlimb, ME; male entire, MN; male neutered, FN; female neutered.

**Figure 6:**
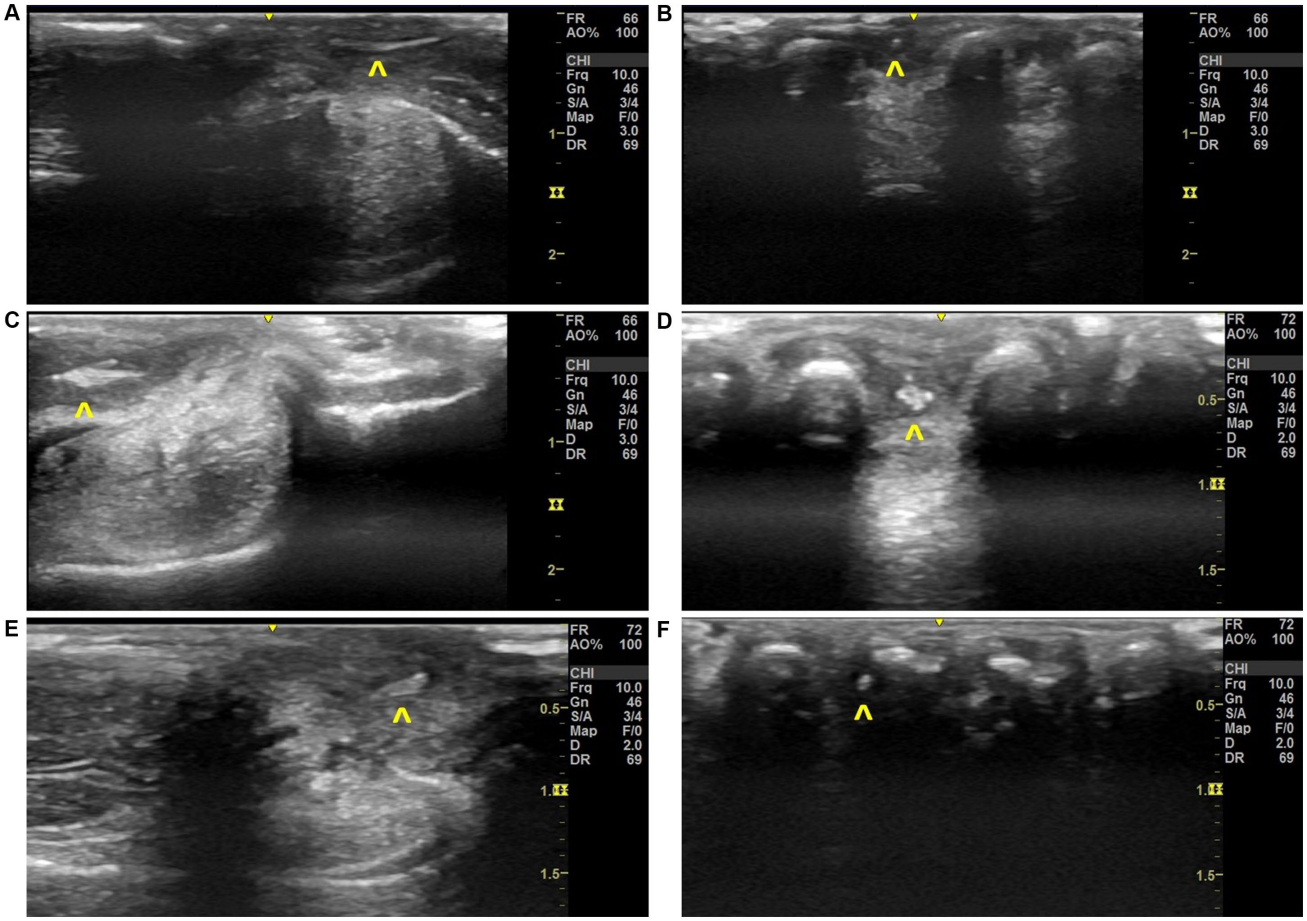
Ultrasonographic images of grass seed vegetal foreign bodies found in local clinical cases, **(A)** Step 11 image with VFB observed in Case 3, **(B)** Step 9 image with VFB observed in Case 3, **(C)** Step 13 image with VFB observed in Case 6, **(D)** Step 9 image with VFB observed in Case 6, **(E)** Step 11 image with VFB observed in Case 8, **(F)** Step 9 image with VFB observed in Case 8. Annotations: 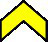, vegetal foreign body.

Using the musculoskeletal protocol, VFBs were positively identified and removed from 87.5% of patients (*n* = 7/8). Grass seeds were typically (*n* = 8/10 limbs) identified first in Step 9 by their ultrasound appearance which could be described as an abnormal hyperechoic structure surrounded by a hypoechoic region between the phalanges (Figure 6). The suspected VFB was then confirmed by the orthogonal view (Step 11) which revealed the typical grass seed spindle-shape. In some instances (Case 5 and 7), the transducer was placed once more in Step 1 to improve visualization of the hyperechoic VFB. In contrast, the ultrasonographic appearance of the VFB varied with Case 8 (Table 1). The VFB could be described as a hyperechoic triangle surrounded by a hypoechoic region which upon removal and review of the patient’s history, was identified as a rose thorn (Figures 6E,F).

A total of 13 VFBs were removed from nine distal limbs. In all patients excluding Case 7, a single VFB was identified on ultrasound. Located just proximal to the localized swelling in all cases, the VFB was removed via ultrasound-guided retrieval and the region re-assessed post-removal. In Case 7, a total of seven VFBs were identified and retrieved from the dog using the described ultrasound method. Two VFBs were identified on ultrasound in the left forelimb (both between the III and IV digits), three from the left hindlimb (one between the IV and V digit, and two between the III and IV digits) and two from the right hindlimb (both located between the I and II digits). In all cases where VFB(s) were removed, no follow-up appointments were made, and each client visited the clinic at least within 12 months of the VFB retrieval for unrelated reasons.

No VFB was identified in just one (*n* = 1/8) patient (Table 1; Case 4). For this case, the presenting history was complex with several differential diagnoses suspected. Historically, a VFB was retrieved blindly from the same limb 3 weeks prior to current presentation and the patient had been used for hunting overnight resulting in a differential diagnosis list that included chronic VFB, remnant fragments from the removed VFB, acute VFB and acute traumatic hunting injury which consequently, resulted in low confidence determining if the case was a true negative. In this instance, no exploration of the wounds was indicated by the ultrasound examination consequently resulting in the distal limb being scrubbed with an antiseptic and the patient sent home with 5 days of antibiotics and anti-inflammatories. No 7-day follow-up appointment was required for this patient indicating resolution of clinical signs.

## 4 Discussion

This is the first reported study detailing the development and application of a method for ultrasonographic examination of the canine lower limb, specifically for the detection of VFBs. The presented methodology study highlights that both a linear transducer and high frequency small-footprint linear array transducer can provide adequate ultrasound images of the ROI and that VFBs can be positively identified when applied to clinical cases. Research has already investigated the use of ultrasonography to assess the ligaments and joints of the dorsal canine carpus. While there are reports on the use of ultrasound to detect grass seeds within the canine limb, there are no studies that have reported a protocol that can be systematically utilized to assess the entire distal limb in a manner that is also applicable in identifying cases with multiple VFBs, complex VFB migration patterns, and VFB location, especially when the site of inflammation is unrelated to the actual VFB location.

The reported SUEDVEG method begins at the interdigital webbing. This is because VFBs have been reported most commonly (74%) between the digits, followed by the carpals (11%), the metacarpals (5%), the metatarsals (5%) and the tarsals (5%) regions (12). Furthermore, it is hypothesized that the more proximal VFBs enter through the webbing, there will be an increased likelihood that the VFB will track proximally through the limb (8, 12, 26, 27). Comparing this to our clinical cases, VFBs were identified within the digital webbing (i.e., step 1 and 2) of just four (*n* = 10) limbs. Vegetal foreign bodies, however, were mostly identified in steps 9 and 11 which is described anatomically as just proximal to the digital webbing. This highlights the fact that grass seeds once entered, can translocate to unexpected areas, and supports the need of a systematic ultrasound protocol to identify VFBs.

Within this study, two transducer frequencies (18 MHz and 12 MHz) were selected to develop the protocol. It was observed that the use of the 18 MHz small-footprint linear array transducer produced enhanced resolution and therefore better visualization of the ROI however, the 12 MHz linear transducer, set at 10 MHz, was more than adequate in all 13 steps. Comparing the general practice, many practitioners will have access to just small curvilinear transducers. This ultrasound protocol should improve the ability to discover VFB with any transducer type. Many curvilinear probes have poor resolution in the near field and therefore it is recommended that clinics with a high incidence of VFB cases considering the use of this ultrasound protocol in practice, invest in a high frequency (>10 MHz) linear transducer. Furthermore, while not used in the development of this protocol, an ultrasound gel offset may be beneficial to improve near field resolution and probe contact. This will therefore improve visualization of the shallow, curved anatomical structures of the distal limb, however, using an offset will not be useful if ultrasound guided retrieval of the VFB is to be attempted.

Probe placement is essential to ensure image quality. Wherever possible, the transducer must be positioned perpendicular to the ROI to prevent artificial decreases in echogenicity of ligaments and tendons seen with acute (< 90^°^) transducer angles (2, 4). Furthermore, anatomical placement must be checked to ensure that sesamoids (Figure 5) are not being incorrectly identified as VFBs and vice versa. This is particularly important on the cranial surface (Steps 9–12) and palmar surface, (Steps 3, 4 and 7). Step 13 (Figure 4) must also be completed with flexion of the digits and pressure on the transducer. Failure to adequately flex the digits may lead to missing pathology underneath the metacarpal/metatarsal pad.

Ultrasonography is a dynamic tool and therefore requires the settings to be regularly adjusted based upon the patient. During any ultrasound examination, the operator must change the frequency, image depth, focus point(s), time gain compensation and gain to optimize the image. In this study, during ultrasound examination, minimal changes were required. Optimal visualization of the ROI was observed at the smallest depth (2 cm) achievable on the ultrasound both in the cadavers and clinical cases due to deeper evaluation not being possible (the varying acoustic impedance between soft tissues and the bone cortex prevents visualization past the bones surface). The focus points were continually adjusted to optimize lateral resolution however due to the superficial nature of the ROI, image quality appeared to be best with two focal points; one at 0.5 cm and 1 cm depth.

Preliminary validation of the systematic ultrasound protocol on a small subset of dogs, resulted in positive identification of VFB(s) in the majority of cases (*n* = 7/8 cases). Twelve grass seed VFB and one rose thorn VFB were identified from 10 dog limbs using the previously reported ultrasonographic grass seed VFB description (28–30). In this study, all VFBs (12 grass seeds and one rose thorn) were hyperechoic with the rose thorn a more triangular shape comparatively to the spindle shape of the grass seeds. All VFBs were surrounded by a hypoechoic region that did not have acoustic shadowing. Furthermore, upon ultrasound guided retrieval, no follow up appointment was required for any patient, leading us to assume that the presenting clinical signs resolved within 7 days.

A mixture of simple and complex VFB cases were assessed using the SUEDVEG method. For most of the cases (*n* = 6/8), completing a focused examination at the site of localized swelling would likely have been quicker and resulted in the same positive identification of a VFB on ultrasound. However, when assessing the two complex cases; Case 4 and 7, the use of a systematic approach was essential. In the instance of Case 4, the generalized swelling and broad history demonstrated that the entire distal limb needed to be systematically examined to be confident that it was a true negative. Furthermore, in the instance of Case 7, multiple VFBs were identified on ultrasound within all three limbs. Case 7 on presentation, was initially presumed to be a simple case and therefore finding multiple VFBs (one of which was unrelated to a draining tract) resulted in it being classified as a complex case part-way through the systematic ultrasound examination. For this reason, it is therefore recommended that a systematic approach as described in this methodology be used in addition to identifying swelling and draining tracts. Clinical signs such as draining tracts and inflammation should be used as a tool to determine the likelihood of where a VFB would track and therefore facilitate the identification of SUEDVEG protocol steps that require extensive evaluation.

The use of a systematic ultrasound protocol reduces the need for operators to find and follow draining tracts. Previously reported as a clinical sign in 53% of cases, draining tracts when observed, have been sonographically followed to locate canine distal limb VFBs (12). While an easy clinical sign to identify and present in 100% of our cases, author experience has found that focused examination techniques have the potential to increase the likelihood of false negative diagnosis. Furthermore, focal diagnostic imaging such as this has the potential to not fully characterize the nature and extent of VFB disease, particularly in the instance of multiple seeds (Table 1; Case 7). Much like the Jamadar et al. (24) study, clinical signs and referred pain can be misleading as seeds can be present at sites with no clinically apparent inflammation or not directly connected to a draining tract. This specific finding was observed in Case 7 where there was no obvious inflammation or draining tract noted between the IV and V digits on the left hindlimb but a VFB was identified proximal to the digits on ultrasound. This therefore suggests that a focal examination is not enough in complex cases and a systematic approach will be more beneficial for VFB diagnosis.

There are several limitations with this study. Firstly, this study has been developed in response to author experience noting that VFB disease commonly recurs despite the use of focused ultrasonographic examinations. The developed protocol incorporates clinical observations, current literature, and the known movement of VFBs through the canine distal limb tissues. However, it has only been utilized on eight patients or 10 limbs. While the results associated with the application of the musculoskeletal protocol to a small subset of clinical cases are promising, this study requires a larger cohort of cases to validate the use of the 13 step ultrasonographic protocol. Secondly, the protocol has not be tested on different ultrasound machines. Veterinary clinics will each have their own brand of ultrasound which will likely require different equipment settings. Low frequency transducers provide poorer resolution images resulting in increased potential to miss minute yet fundamental pathology. Furthermore, certain ultrasound machine brands may function best at different focus point(s), dynamic range, time gain compensation and gain. Clinics will need to investigate what ultrasound settings provide the best ultrasound image for them. Future investigations need to include a wider number and variety of dog breeds to characterize what a veterinarian will observe in a clinical setting and utilize a variety of ultrasound machines to therefore validate the presented musculoskeletal ultrasound protocol.

The purpose of this paper was to establish a standardized ultrasound protocol for the systematic examination of the canine distal limb for the identification of VFBs. This study demonstrates that the SUEDVEG method was successful in identifying grass seeds in the majority of cases evaluated with resolution of clinical signs within all 10 limbs. Further research is required to evaluate the use of this ultrasound methodology compared to other forms of distal limb VFB identification.

## Data availability statement

The raw data supporting the conclusions of this article will be made available by the authors, without undue reservation.

## Ethics statement

The animal studies were approved by Charles Sturt University Animal Ethics Committee. The studies were conducted in accordance with the local legislation and institutional requirements. Written informed consent was obtained from the owners for the participation of their animals in this study.

## Author contributions

ES: Conceptualization, Methodology, Validation, Writing – original draft, Writing – review & editing. MC: Conceptualization, Investigation, Methodology, Supervision, Validation, Writing – review & editing. EC: Investigation, Validation, Writing – review & editing. KJ: Conceptualization, Methodology, Supervision, Writing – review & editing. RR: Conceptualization, Investigation, Methodology, Supervision, Writing – review & editing.
